# Relationship between exposure to the *Avahan* intervention and levels of reported condom use among men who have sex with men in southern India

**DOI:** 10.1186/1471-2458-14-1245

**Published:** 2014-12-04

**Authors:** Kate M Mitchell, Anna M Foss, Banadakoppa M Ramesh, Reynold Washington, Shajy Isac, Holly J Prudden, Kathleen N Deering, James F Blanchard, Stephen Moses, Catherine M Lowndes, Marie-Claude Boily, Michel Alary, Peter Vickerman

**Affiliations:** Department of Global Health and Development, London School of Hygiene and Tropical Medicine, London, UK; Karnataka Health Promotion Trust, Bangalore, India; Department of Community Health Sciences, University of Manitoba, Winnipeg, Canada; Department of Medicine, University of British Columbia, Vancouver, Canada; Centre for Global Public Health, University of Manitoba, Winnipeg, Canada; Public Health England, London, UK; Centre de recherche du CHU de Québec, Québec, Canada; Department of Infectious Disease Epidemiology, Imperial College London, London, UK; School of Social and Community Medicine, University of Bristol, Bristol, UK

**Keywords:** Consistent condom use, Condom use at last sex act, Condom demonstration, Key population, Bangalore, Cross-sectional study, Logistic regression

## Abstract

**Background:**

The *Avahan* intervention promotes consistent (100%) condom use amongst men who have sex with men in southern India. We assessed how condom use varies with intervention exposure for men who have sex with men in Bangalore.

**Methods:**

Self-reported condom use and intervention exposure data were derived from a cross-sectional survey. Consistent condom use and condom use at last sex act with all, main, and casual male sex partners were assessed. Binary and continuous variables reflecting intervention exposure (including contact(s) with intervention staff, receiving condoms and seeing condom demonstrations) were used. Multivariable logistic regression was employed to assess the relationship between condom use with each type of partner and each exposure variable independently, controlling for socio-demographic and behavioural factors associated with condom use or intervention exposure.

**Results:**

Condom use with all partners was higher among those who had ever been contacted by, received condoms from, or seen a condom demonstration by intervention staff (adjusted odds ratio >2, p < 0.02 for all). Consistent condom use with all types of partner increased with the number of condom demonstrations seen in the last month (adjusted odds ratio = 2.1 per demonstration, p < 0.025), while condom use at last sex act with a casual (but not main) partner increased with the number of condoms received from the intervention (adjusted odds ratio = 1.4 per condom, p = 0.04).

**Conclusions:**

Direct contact with *Avahan* program staff is associated with increased reported condom use among men who have sex with men in Bangalore. Reported consistent condom use and condom use at last sex act are associated with contacts involving demonstrations of correct condom use, and with receiving condoms, respectively.

## Background

Condoms are highly protective against HIV transmission when used consistently and correctly [1–4]. The India AIDS Initiative (*Avahan*) of the Bill & Melinda Gates Foundation, an HIV prevention program which targets populations at high risk of HIV acquisition in the highest HIV prevalence states in India, aims to directly reduce HIV transmission among and from these high-risk groups by treating bacterial sexually transmitted infections (STIs) and promoting consistent condom use (CCU) [5, 6]. One of the high-risk groups targeted is men who have sex with men (MSM), a marginalised group in southern India, known to have high HIV prevalence (7-21% in different districts of Andhra Pradesh, Maharashtra, Tamil Nadu and Karnataka states [7–10]). Surveys of MSM across southern India have suggested high levels of engagement in commercial sex [7–9], with 40-68% of MSM in different surveys reporting ever receiving payment for sex [7, 9]. Much smaller numbers of MSM (3-7%) report sex work as their main source of income, excepting Karnataka state where 57% of MSM surveyed reported sex work as their main income source [7, 10]. It should be noted that these surveys tend to capture higher-risk MSM, and so they are likely to overestimate the true proportion of MSM who sell sex [11].

Evaluation of the impact of the *Avahan* intervention has been challenging; there is no control group (for ethical and logistical reasons), and baseline surveys were carried out some time after the intervention started [6, 12, 13]. Several approaches have been used to estimate how *Avahan* has affected condom use [13–18]. One analysis found that in Karnataka state, reported levels of CCU by female sex workers (FSWs) with their commercial clients was higher among FSWs who reported contact with the *Avahan* intervention [16]. It also found that condom use increased with time since initial contact with the intervention, with increased numbers of contacts with program outreach staff, and (more strongly) with the number of condom demonstrations witnessed, in a dose-dependent manner [16]. Such analyses can be used to inform calculations of the cost-effectiveness of different intervention options, for example the relative impact of more intense interventions reaching fewer high-risk individuals compared with less intense interventions reaching more people [19].

Here, we performed a similar analysis for self-reported condom use among MSM in Bangalore, southern India, to see whether contact with the *Avahan* intervention was linked to an increased level of self-reported condom use with different male sexual partners. In Bangalore, *Avahan* services were delivered to MSM by a local non-governmental organisation (NGO), *Sangama. Sangama* had been working with local MSM for some years previously, promoting sexual minority rights, and had been delivering *Avahan* HIV-prevention services for about eleven months prior to the survey used here.

Since the previous study amongst FSWs suggested that different exposure measures, reflecting different aspects of the intervention, may vary in their relationship with condom use [16], we looked at a number of different measures of intervention exposure to identify the particular components of contact with the *Avahan* intervention which were associated with increased self-reported condom use by MSM.

## Methods

### Data

The data come from a cross-sectional Integrated Biological and Behavioural Assessment (IBBA), which was conducted in urban Bangalore, Karnataka in 2006 as part of the evaluation of *Avahan* [5, 7, 11]. Respondents provided blood samples for HIV and STI testing, and provided behavioural information through a structured face-to-face questionnaire. In Bangalore, since the *Avahan* intervention is delivered by local NGO *Sangama*, all questions about contact with *Avahan* refer to contact with *Sangama*. MSM were recruited by time-location sampling at local ‘cruising sites’ (locations where men are known to look for male sexual partners) [11]. Three hundred and twenty out of 554 MSM approached (58%) completed the behavioural survey [20] and are included in this analysis. Standardized weights were assigned to the data to account for differences in selection probabilities between different clusters of MSM arising from the sampling design [7, 11]; clusters were also taken into account, using the Complex Samples module in SPSS.

This research received approval from the Ethics Committee of the London School of Hygiene and Tropical Medicine. The Bangalore data collection methods were approved by the Ethics Review Boards of St Johns Medical College and Hospital, Bangalore, the Centre hospitalier affilié universitaire de Québec (CHA), Québec, Canada, and the University of Manitoba, Canada. For the monitoring and evaluation of the Avahan intervention in India, ethical approval was also obtained from the Ethics Review Board of the Centre hospitalier affilié universitaire de Québec, Québec, Canada.

### Outcome variables

The outcome variables were: (1) reporting CCU (study participants responded that in general they used condoms “every time” rather than “most of the time”, “sometimes”, or “never”); and (2) reporting using a condom at last sex act, with each of the following types of partner: (i) all male sexual partners; (ii) main (regular) male sexual partner; (iii) casual (“new” or “unknown”) male sexual partners. Only MSM who reported having sex with new or unknown partners in the past week were asked about condom use with casual partners in the survey. Although many MSM reported selling sex, the data collected in this survey did not differentiate between commercial and non-commercial partners, and so both ‘all’ and ‘casual’ partners are likely to reflect a mixture of commercial and non-commercial partnerships.

### Exposure variables

Three binary variables reflecting exposure to the *Avahan* intervention were investigated: whether or not MSM had ever been contacted by community mobilizers/staff from *Sangama*; whether or not they had ever been given condoms by *Sangama* staff; and whether they had ever seen a demonstration of correct condom use by a *Sangama* worker. Four continuous exposure variables were also investigated: duration since first contact by *Sangama* staff (for those ever contacted); number of times contacted by *Sangama* staff in the last month (for those ever contacted); number of condoms received on the most recent occasion that they were given condoms by *Sangama* staff (for those who had ever received condoms); and number of condom demonstrations seen in the last month (for those who had ever seen a condom demonstration).

### Socio-demographic and behavioural variables

A number of additional variables which might influence the relationship between intervention exposure and condom use were also examined for any relationship with either condom use or intervention exposure. All of the continuous variables were also considered as categorical variables, which were derived by dividing the data into quartiles, giving four groups of roughly equal sizes. The variables considered were: age (continuous variable or categorised into 16-22/23-25/26-31/32-60 years of age); duration as an MSM (calculated as time since first had sex with a man; continuous variable or categorised into 0-4/5-7/8-13/14+ years); ever married to a woman; ever paid a female for sex; religion (Hindu or other (Muslim or Christian)); circumcision status; literacy (whether or not they could read and write); school grade reached for those literate (categorised into 0-8/9-10/11-12/13-16); had ever sold sex; sex work being their main source of income; three sexual identity groups (*Kothis* + *Hijras*/*Panthis* + bisexuals/double deckers (*Kothis* and *Hijras* mainly take the receptive role in anal sex, *Panthis* and bisexuals mainly take the insertive role, and double deckers take both roles [21–23]); location where sex usually occurs with male partners (public or private, where public = bar/nightclub, public garden, public toilet, railway station, bus stop/stand, cinema hall/theatre or other public place, and private = home, rented room, vehicle, hammam or lodge); number of MSM partners having had anal sex with in the last week (continuous variable or categorised into 0/1-2/3-4/5+); number of times having had anal sex with known partners in the last week (continuous variable or categorised into 0/1-2/3/4+); and the number of times having had anal sex with new partners in the last week (continuous variable or categorised into 0/1-2/3-5/6+).

### Statistical analysis

Logistic regression analyses were used to identify the factors associated with each of the outcome variables (condom use with each partner type, either at last act or consistently). First, we looked separately at the relationship between each outcome variable and each of the intervention exposure variables in univariate regression analyses. Second, we identified any socio-demographic and behavioural variables which were associated with either the outcome or the intervention exposure variables (i.e. potential confounding variables) using univariate regression – logistic regression for assessing associations with condom use variables and binary exposure variables, and linear regression for assessing associations with continuous exposure variables.

Separate multivariable logistic regression models were constructed for each outcome variable and each exposure variable, which included relevant confounding variables. Socio-demographic or behavioural variables were considered for inclusion in the multivariable models if the p-value for their association with either the exposure or the outcome variable was less than 0.1. Since the variables reflecting sexual activity (number of partners in the last week, or number of sex acts with regular or new partners in the last week) were highly correlated, we identified the one which was most often associated with condom use – number of sex acts with new male partners in the last week (as a continuous variable) - and included this (and none of the other sexual activity variables) in all multivariable models. Since ‘sex work as main income source’ is a subset of ‘ever sold sex’, when both of these variables were associated with the outcome, the one most closely associated was included in multivariable models. All variables were entered simultaneously into the multivariable models.

Results were considered to be statistically significant if p < 0.05. All associations of the main exposure and outcome variables with socio-demographic and behavioural variables with a p-value < 0.1 are reported, as these variables were considered for inclusion in multivariate models.

All analyses were performed using IBM SPSS Statistics version 20.0.0.

## Results

### Population characteristics

The MSM surveyed had a mean age of 28 years old (range 16-60), and had been having sex with men for an average of 10 (range 0-37) years. Forty-one percent reported sex work as their main source of income, 76% were literate and 82.5% were Hindu. Fifty-one percent of the sample self-identified as *Kothi* or *Hijra*, 22% as *Panthi* or bisexual and 27% as double deckers. One hundred and thirty-one MSM (41%) reported a main male sexual partner and 238 (76%) declared casual male partners. MSM reported an average of three (range 0-50) male sex partners in the past week. Twenty-four percent had ever been married to a woman, and 16% had ever paid a female for sex.

### Intervention exposure

Seventy-two percent of MSM reported ever being contacted by *Sangama* staff, 67% had received condoms from *Sangama*, and 56% had witnessed a condom demonstration. There was considerable overlap between these exposures; all of those who received condoms or seen a condom demonstration had also been contacted by the intervention, and 55% of MSM reported all three of these exposures (they had been contacted, received condoms and seen a condom demonstration). Almost all (98%) of those who had seen a condom demonstration also reporting receiving condoms. Among those contacted, there was a positive correlation between duration since first contact with *Sangama* and number of contacts in the last month (r = 0.185, p = 0.005); this association persisted when only those who had first had contact with *Sangama* within the previous 11 months (since *Sangama* began offering *Avahan* services) were considered (r = 0.211, p = 0.03, n = 106). No association was seen between the other continuous intervention exposure variables.

#### Associations with socio-demographic and behavioural variables

Men who had ever been contacted by *Avahan* were more likely to have ever sold sex than those who had never been contacted (odds ratio (OR) = 2.17, p = 0.010). MSM who had ever (versus never) received condoms from *Avahan* were more likely to have ever sold sex (OR = 2.23, p = 0.006) and less likely to have ever had sex with a female sex worker (OR = 0.51, p = 0.083). Men who had ever (versus never) seen a condom demonstration were also more likely to have ever sold sex (OR = 2.26, p = 0.005), and less likely to have ever had had sex with a female sex worker (OR = 0.50, p = 0.038). Number of contacts with the intervention in the past week was associated with duration as an MSM (lowest for those who had been MSM for 8-13 years, highest for those who had been MSM for 14+ years), and was lower for those with sex work as their main income (1.5 fewer contacts per month, p = 0.07). More condoms were received by those who had never paid a female for sex (versus ever; 56 more condoms, p = 0.05), by those who had ever sold sex (94 more condoms, p = 0.03), by those for whom sex work was their main source of income (149 more condoms, p = 0.03), and by *Kothis* and *Hijras* (versus other subgroups; 130 more condoms; p = 0.01). More condom demonstrations were reported by those who had never been married to a woman (versus ever; 1.4 more demonstrations per month, p = 0.03), those who had never paid a female for sex (1.2 more per month, p = 0.07), and by double deckers (2.6 more per month), and *Kothis* and *Hijras* (1.5 more per month) (versus *Panthis* and bisexuals; p = 0.002).

### Factors associated with CCU

Seventy-one percent of MSM reported CCU with all male partners, 74% reported CCU with their main male partner, and 74% reported CCU with casual male partners.

#### Associations with socio-demographic and behavioural variables

CCU with all partners was related to duration as an MSM (highest among those who had been MSM 5-7 years; p = 0.051) and was higher among those who had ever sold sex (p = 0.003), those with sex work as their main income (p < 0.001), and *Kothi* and *Hijra* (p = 0.004). CCU with causal partners was also higher among those who had ever sold sex (p = 0.01), those with sex work as their main income (p < 0.001), and among Kothi and Hijra (p = 0.014), and was also higher amongst those having sex in private (versus public) locations (OR = 2.0, p = 0.08). CCU was more closely associated with sex work as main income than ever selling sex, so ‘sex work as main income’ was included in multivariable models. Less CCU with main partners was reported by Hindus (OR = 0.37, p = 0.08), and levels of CCU with main partners increased with increasing number of sex acts with new partners in the last week (OR per additional partner = 1.3, p = 0.01).

#### Unadjusted and adjusted associations with intervention exposure

**Figure 1:**
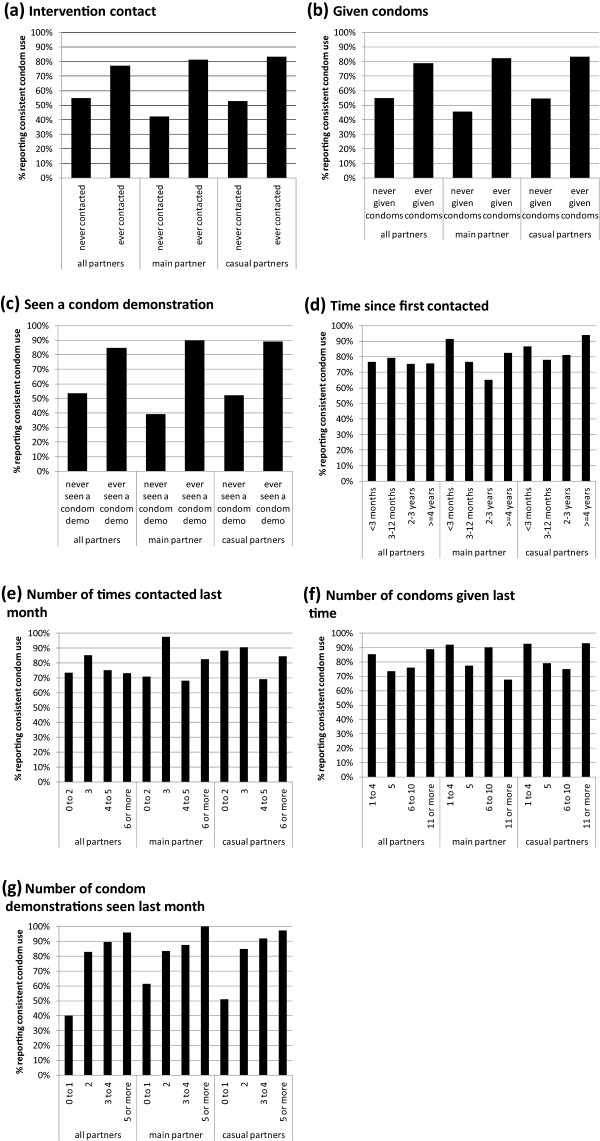
**Levels of reported consistent condom use (CCU) by intervention exposure.** CCU is shown with the following different partner types: all, main, casual male sexual partners. CCU is shown for MSM with the following intervention exposures: **(a)** ever contacted by intervention staff versus not; **(b)** ever received condoms from the intervention versus not; **(c)** ever witnessed a condom demonstration versus not; **(d)** duration since first contacted by the intervention; **(e)** number of times contacted by the intervention in the last month; **(f)** number of condoms received from the intervention the last time that they were given condoms; **(g)** number of condom demonstrations seen in the last month.

In unadjusted analyses, CCU with all partners was significantly higher among those who had been contacted by the *Avahan* intervention (77% versus 55%), those who had received condoms from the intervention (79% versus 55%) and those who had seen a condom demonstration (85% versus 54%) (Figure 1a-c). Even greater differences were seen between the exposed and unexposed MSM in CCU with specific partner types for each of these binary measures (Figure 1a-c). All of these associations remained significant after adjusting for socio-demographic and behavioural factors which were associated with either CCU or with intervention exposure (Table 1). CCU was not associated with duration since first intervention contact or number of contacts with the intervention in the past month in either unadjusted or adjusted analysis (Figure 1d,e, Table 1). In unadjusted analysis, no relationship was seen between CCU and number of condoms received, but after adjusting for potential confounders, CCU with the main partner only was found to decrease slightly with increasing number of condoms received (adjusted odds ratio (AOR) = 0.994, p < 0.001). CCU with all partner types increased with the number of condom demonstrations seen in the last month in both unadjusted (Figure 1g) and adjusted analyses (for each additional condom demonstration, AOR = 2.15 for all partners (p = 0.003), 2.11 for main partner (p = 0.014), 2.06 for casual partners (p = 0.024); Table 1).

**Table 1:** **Associations between intervention exposure variables and consistent condom use with different partners**

	CCU with all partners (n = 305)*				CCU with main partner (n = 122)*				CCU with casual partners (n = 235)*			
	OR ^a^(95% CI)	p-value	AOR ^b,c^(95% CI)	p-value	OR ^a^(95% CI)	p-value	AOR ^b,d^(95% CI)	p-value	OR ^a^(95% CI)	p-value	AOR ^b,e^(95% CI)	p-value
Intervention exposure												
Ever contacted (versus never)^f^	**2.76 (1.41-5.40)**	**0.004**	**2.65 (1.27-5.52)**	**0.010**	**5.92 (1.58-22.23)**	**0.009**	**8.48 (1.90-37.9)**	**0.006**	**4.43 (1.95-10.1)**	**0.001**	**6.45 (2.96-14.1)**	**<0.001**
Ever given condoms (versus never)^g^	**3.10 (1.66-5.79)**	**0.001**	**3.15 (1.60-6.22)**	**0.001**	**5.50 (1.57-19.33)**	**0.009**	**7.90 (1.69-37.0)**	**0.010**	**4.14 (1.86-9.2)**	**0.001**	**5.75 (2.56-12.9)**	**<0.001**
Ever seen a condom demo (versus never)^h^	**4.75 (2.60-8.68)**	**<0.001**	**4.24 (2.23-8.07)**	**<0.001**	**13.67 (4.23-44.17)**	**<0.001**	**8.72 (2.24-34.0)**	**0.002**	**7.43 (3.30-16.7)**	**<0.001**	**7.32 (3.26-16.4)**	**<0.001**
Duration since first contact (per year)	0.96 (0.79-1.17)	0.707	0.87 (0.70-1.08)	0.194	0.91 (0.69-1.20)	0.491	0.89 (0.71-1.11)	0.294	1.06 (0.77-1.46)	0.706	0.90 (0.65-1.26)	0.540
Number of contacts last month (per contact)^i^	0.96 (0.91-1.01)	0.115	0.97 (0.92-1.03)	0.313	1.05 (0.95-1.16)	0.344	1.07 (0.94-1.21)	0.291	0.97 (0.92-1.04)	0.391	0.98 (0.91-1.05)	0.478
Number of condoms given last time (per condom)^j^	1.00 (1.00-1.00)	0.424	1.00 (1.00-1.00)	0.956	1.00 (0.99-1.00)	0.277	**0.994 (0.992-0.997)**	**<0.001**	1.01 (0.99-1.02)	0.281	1.01 (0.99-1.02)	0.304
Number of condom demos seen last month (per time)^k^	**1.97 (1.30-2.97)**	**0.002**	**2.15 (1.30-3.54)**	**0.003**	**2.31 (1.47-3.63)**	**0.001**	**2.11 (1.18-3.78)**	**0.014**	**2.13 (1.32-3.45)**	**0.003**	**2.06 (1.10-3.86)**	**0.024**

Odds ratios which are significantly different from 1 are shown in bold (p < 0.05).

*Unadjusted number of people answering questions about condom use with each partner type. Number of individuals included in each analysis may be smaller, depending upon whether they answered questions relating to the intervention exposure under analysis; for continuous exposure variables, unexposed individuals are excluded.

^a^OR are unadjusted odds ratios.

^b^AOR are adjusted odds ratios, adjusted for the factors listed below.

^c^Adjusted for duration as MSM (categorised), sex work as main income, MSM identity (in 3 groups) and number of times had anal sex with new partners last week.

^d^Adjusted for religion (Hindu versus other) and number of times had anal sex with new partners last week.

^e^Adjusted for sex work as main income, MSM identity (in 3 groups), location where usually have sex with male partners (public or private), and number of times had anal sex with new partners last week.

^f^AOR adjusted for whether ever sold sex.

^g^AOR adjusted for whether ever paid a female for sex and whether ever sold sex.

^h^AOR adjusted for whether ever paid a female for sex and whether ever sold sex.

^i^AOR adjusted for duration as MSM (categorised) and sex work as main income.

^j^AOR adjusted for whether ever paid a female for sex, sex work as main income and MSM identity (in 3 groups).

^k^AOR adjusted for whether ever married to a woman, ever paid a female for sex and MSM identity (in 3 groups).

### Factors associated with condom use at last sex act

The percentage of MSM reporting condom use at last sex act was 79% for sex with any male partner, 78% with the main partner and 86% with casual partners.

#### Associations with socio-demographic and behavioural variables

Condom use at last sex with any partner was lower among those who had ever been married to a woman (OR = 0.48, p = 0.06), higher among those who had ever sold sex (p = 0.004), higher for those for whom sex work was their main source of income (p = 0.04), and increased significantly with each additional sex act with casual partners in the past week (OR = 1.31, p = 0.016). Condom use at last sex act with the most recent casual partner was higher amongst those who had ever sold sex (p < 0.001) and amongst those for whom sex work was their main source of income (p = 0.044). Condom use at last sex act was more closely associated with ever selling sex than with sex work as main income source, so ‘ever sold sex’ was included in multivariable models. Condom use at last sex with the main partner increased significantly with each additional sex act with casual partners in the past week (OR = 1.32, p = 0.02), and was lower among MSM who were Hindus (p = 0.001), circumcised (p = 0.009) or illiterate (p = 0.08).

#### Unadjusted and adjusted associations with intervention exposure

**Figure 2:**
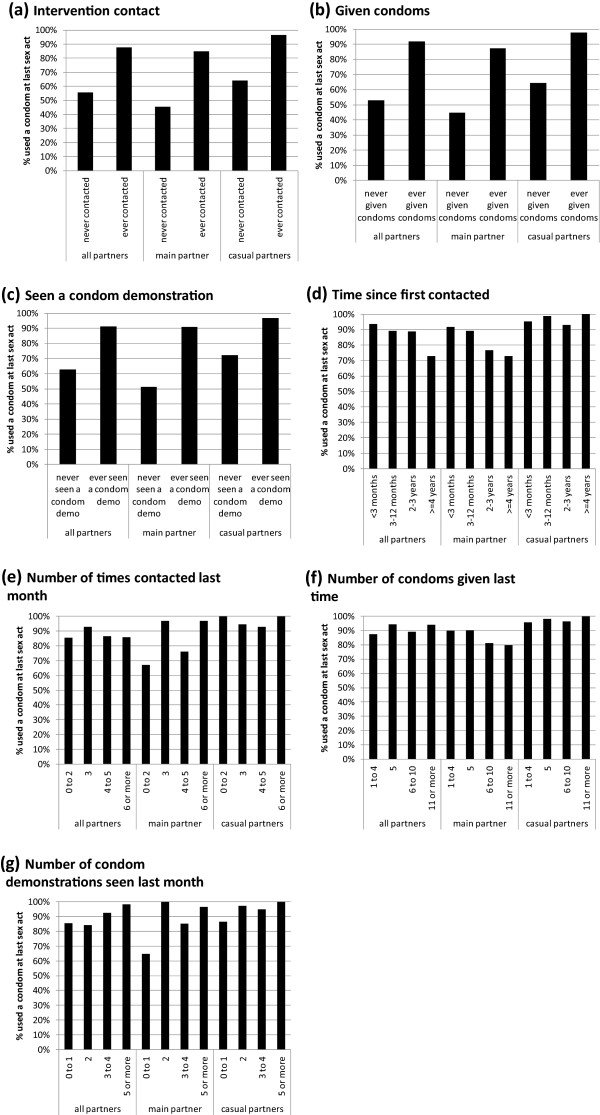
**Proportion of MSM using a condom at last sex act by intervention exposure.** Condom use at last sex act is shown with the following different partner types: all, main and casual male sexual partners. Condom use at last sex act is shown for MSM with the following intervention exposures: **(a)** ever contacted by intervention staff versus not; **(b)** ever received condoms from the intervention versus not; **(c)** ever witnessed a condom demonstration versus not; **(d)** duration since first contacted by the intervention; **(e)** number of times contacted by the intervention in the last month; **(f)** number of condoms received from the intervention the last time that they were given condoms; **(g)** number of condom demonstrations seen in the last month.

As with CCU, condom use at last sex act with all partner types was significantly higher among those ever contacted by the intervention, those ever receiving condoms from the intervention, and among those ever seeing a condom demonstration, in both unadjusted and adjusted analyses (Figure 2a-c, Table 2). Adjusting for socio-demographic and behavioural factors revealed a slight decrease in condom use at last sex with main partners with increasing numbers of condoms received (Table 2). Unlike CCU, a negative association was seen between duration since initial contact with the intervention and condom use at last sex act with both any and main partners (Figure 2d, e), and this was significant for condom use at last sex act with any partner in adjusted analysis (Table 2). Unlike with CCU, in adjusted analysis, none of the condom use at last sex act variables were significantly associated with number of condom demonstrations seen, and following adjustment for confounders, condom use at last sex act with casual partners was found to increase with the number of condoms received (AOR = 1.35, p = 0.04; Table 2).

**Table 2:** **Associations between intervention exposure variables and condom use at last sex act with different partners**

	Condom use last sex act with any partner (n = 303)*				Condom use last sex act with main partner (n = 131)*				Condom use last sex act with a casual partner (n = 238)*			
	OR ^a^(95% CI)	p-value	AOR ^b,c^(95% CI)	p-value	OR ^a^(95% CI)	p-value	AOR ^b,d^(95% CI)	p-value	OR ^a^(95% CI)	p-value	AOR ^b,e^(95% CI)	p-value
Intervention exposure												
Ever contacted (versus never)^f^	**5.82 (2.49-13.6)**	**<0.001**	**7.55 (3.31-17.2)**	**<0.001**	**7.15 (2.63-19.4)**	**<0.001**	**6.75 (1.66-27.34)**	**0.008**	**15.1 (6.05-37.5)**	**<0.001**	**12.5 (4.90-32.0)**	**<0.001**
Ever given condoms (versus never)^g^	**10.0 (4.20-24.1)**	**<0.001**	**12.2 (5.32-28.1)**	**<0.001**	**8.48 (3.14-22.9)**	**<0.001**	**5.81 (1.41-24.0)**	**0.016**	**23.1 (7.90-67.5)**	**<0.001**	**21.1 (6.36-70.0)**	**<0.001**
Ever seen a condom demo (versus never)^h^	**6.24 (2.68-14.5)**	**<0.001**	**5.27 (2.41-11.5)**	**<0.001**	**9.61 (3.34-27.6)**	**<0.001**	**6.70 (1.46-30.9)**	**0.016**	**11.0 (4.16-29.0)**	**<0.001**	**8.76 (3.09-24.8)**	**<0.001**
Duration since first contact (per year)	**0.78 (0.63-0.97)**	**0.027**	**0.74 (0.56-0.98)**	**0.037**	**0.76 (0.60-0.97)**	**0.026**	0.85 (0.57-1.26)	0.407	1.16 (0.72-1.87)	0.541	1.15 (0.69-1.92)	0.592
Number of contacts last month (per contact)^i^	0.98 (0.91-1.01)	0.476	0.93 (0.84-1.02)	0.130	1.43 (0.90-2.27)	0.132	1.46 (0.91-2.36)	0.115	**1.12 (1.02-1.24)**	**0.025**	1.11^l^ (0.97-1.27)	0.124
Number of condoms given last time (per condom)^j^	1.00 (1.00-1.00)	0.876	1.00 (1.00-1.00)	0.225	1.00 (0.99-1.00)	0.156	**0.993 (0.988-0.999)**	**0.021**	1.30 (0.81-2.11)	0.272	**1.35 (1.01-1.79)**	**0.040**
Number of condom demos seen last month (per time)^k^	**1.60 (1.07-2.39)**	**0.022**	1.26 (0.75-2.10)	0.378	1.56 (0.90-2.70)	0.107	1.68^m^ (0.62-4.55)	0.301	1.88 (0.93-3.80)	0.080	1.94 (0.81-4.66)	0.134

Odds ratios which are significantly different from 1 are shown in bold (p < 0.05).

*Unadjusted number of people answering questions about condom use with each partner type. Number of individuals included in each analysis may be smaller, depending upon whether they answered questions relating to the intervention exposure under analysis; for continuous exposure variables, unexposed individuals are excluded.

^a^OR are unadjusted odds ratios.

^b^AOR are adjusted odds ratios, adjusted for the factors listed below.

^c^Adjusted for duration as an MSM (categorised), whether ever sold sex, whether ever married to a woman, and number of times had anal sex with new partners last week.

^d^Adjusted for religion (Hindu versus other), circumcision status, literacy (literate versus not) and number of times had anal sex with new partners last week.

^e^Adjusted for whether ever sold sex and number of times had anal sex with new partners last week.

^f^AOR adjusted for whether ever sold sex.

^g^AOR adjusted for whether ever paid a female for sex and whether ever sold sex.

^h^AOR adjusted for whether ever paid a female for sex and whether ever sold sex.

^i^AOR adjusted for duration as MSM (categorised) and sex work as main income.

^j^AOR adjusted for whether ever paid a female for sex, sex work as main income and MSM identity (in 3 groups).

^k^AOR adjusted for whether ever married to a woman, ever paid a female for sex and MSM identity (in 3 groups).

^l^Not adjusted for duration as an MSM, as it overlapped too much with the number of intervention contacts in this subset of the population.

^m^Not adjusted for circumcision status, as it overlapped too much with religion in this subset of the population.

## Discussion

Our analysis shows that direct contact with the *Avahan* intervention was associated with increased reported condom use among MSM in Bangalore. MSM reporting any exposure to the intervention had higher condom use than those who did not. Consistent condom use with all male partner types was highest among MSM who had witnessed a condom demonstration, and increased steadily with the number of demonstrations seen. Condom use at last sex act with casual partners was highest among those who had received condoms from the intervention, and increased with the number of condoms received. Condom use did not increase with duration since initial intervention exposure, nor with more frequent contacts with *Avahan* staff.

The finding that CCU is higher among those exposed to *Avahan* agrees with similar analyses involving FSWs in Karnataka; however, CCU was higher among FSWs who had been exposed to the intervention for longer [16], which was not seen here. This may be because *Sangama*, the NGO delivering *Avahan* services, had been working with MSM for some years previously but only delivering *Avahan* HIV-prevention services for about eleven months prior to the survey so that duration of contact with *Sangama* may not accurately reflect duration of *Avahan* exposure. Although neither factor was associated with condom use, we found that MSM with longer exposure to *Sangama* also reported more frequent contacts, including over the eleven months for which *Sangama* had delivered *Avahan* services. One plausible hypothesis for this association is that MSM who contacted the intervention earlier on were more pro-active and more likely to contact the intervention repeatedly.

The strong association between CCU and the number of condom demonstrations seen was also found among FSWs [16], and contrasts with the lack of positive association between CCU and total number of intervention contacts. In condom demonstrations, MSM are shown and then practise themselves the correct way to unpack condoms and place them on a penis model; condom demonstrations are also used to reinforce risk reduction messages, and to distribute condoms. Thus, a condom demonstration may represent a contact with the program where prevention messages are communicated, as well as giving a direct learning benefit of observing and practising correct condom use [24, 25]. Some HIV risk-reduction models propose that behavioural skills, including correct condom use, are a necessary component for individuals’ HIV risk-reduction [26]. While the FSW analysis suggested that condom use may saturate for those seeing more than two condom demonstrations per month [16], no saturation effect was seen here for MSM.

The number of condoms received was only positively associated with condom use with casual, not main, partners. Similarly, for FSWs in Karnataka, intervention exposure was associated with increased condom use with commercial, but not main, partners [16]. Counter-intuitively, we found a slight decrease in reported condom use (both CCU and condom use at last sex act) with main partners with increasing number of condoms received. While this is of concern, the effect is relatively small (AOR 0.99 per additional condom), and likely to be outweighed by the positive impact on condom use with casual partners, who are more frequently reported by MSM in this sample. Our finding that condom use at last sex act with a casual partner was more strongly associated with receiving condoms from the program than was CCU, is consistent with data from other IBBA surveys among MSM across southern India [7]. CCU reflects higher overall, and perhaps longer-term, condom use, which may be less influenced by recent availability of condoms than condom use at last sex act.

Several studies have found increased condom use following behavioural interventions among MSM [27–31]. In agreement with our findings, studies in China found that condom use was associated with receiving condoms and peer education [32], or with reported contact with a prevention program [33].

The data used in this study were collected eight years ago, and while this means that they may not reflect current behaviour (which may be affected by, for example, increased access to ART), they were collected at a time sufficiently soon after the beginning of the *Avahan* intervention to have a large enough population still unexposed to the intervention allowing for comparisons to be made between exposed and unexposed individuals. In more recent surveys amongst this population [34], the proportion of MSM never contacted by the intervention was greatly reduced, limiting the usefulness of such comparisons.

The high levels of condom use among MSM contacted by the *Avahan* intervention, coupled with the high levels of contact with the intervention reported, suggest that the *Avahan* intervention could have a large impact upon HIV transmission amongst MSM in Bangalore. No significant change in HIV prevalence was found amongst MSM in Bangalore between two sero-prevalence surveys carried out in 2006 and 2009 [34], but this does not necessarily mean that *Avahan* had no impact, as it is possible that HIV prevalence could have risen in the absence of the *Avahan* program.

### Limitations

Our study used self-reported data, which may be subject to social desirability bias. Social desirability bias is likely to have led to an overestimate of the true impact of the intervention upon condom use in this study, since those exposed to the intervention should have heard more messages promoting condom use than those not exposed, and so may feel a greater pressure to report high condom use. On the other hand, the study may have underestimated the true impact of the intervention upon condom use, since those MSM not directly exposed to the intervention may be having sex with those who have been exposed and are using condoms more frequently. There may have been a selection effect by the intervention, i.e. those reached by *Avahan* may have been more likely to use condoms anyway. We attempted to control for this by including in our multivariate models measured factors associated with exposure to the intervention, but there may have been other unmeasured factors which could account for both a higher likelihood of intervention exposure and higher condom use. The sampling methods used to gather the data (designed to capture high-risk MSM), and relatively low participation rate, implies that the data may not be representative of the wider MSM population. The analysis used cross-sectional data, and so we cannot demonstrate whether exposure to *Avahan* preceded increased levels of condom use.

### Recommendations

In light of our findings, we recommend that the *Avahan* program, and other programs following this model, focus upon providing high-quality contacts with MSM, which wherever possible should include demonstrations of correct condom use and distribution of condoms, as well as communication about risk reduction.

## Conclusions

Reported condom use rates by MSM in Bangalore were higher among those who had been contacted by the *Avahan* intervention. Consistent condom use was associated with repeated contacts with the program, which involved demonstrations of correct condom use, while condom use at last sex act with casual partners was associated with recently receiving condoms from the program.

## Abbreviations

AOR: Adjusted odds ratio
CCU: Consistent condom use
FSWs: Female sex workers
MSM: Men who have sex with men
NGO: Non-governmental organization
OR: Odds ratio
STIs: Sexually transmitted infections.
